# Impact of BMI on basal LH in premenarcheal girls with idiopathic central precocious puberty

**DOI:** 10.3389/fendo.2025.1740527

**Published:** 2026-01-07

**Authors:** Xin Yuan, Ying Zhang, Jing Zhang, Wenyong Wu, Ruimin Chen

**Affiliations:** 1 Department of Endocrinology, Genetics and Metabolism, Fuzhou First General Hospital Affiliated with Fujian Medical University, Fuzhou Children’s Hospital of Fujian Medical University, Fuzhou, China; 2 Department of Pediatrics Clinical Medicine, Quanzhou Medical College, Quanzhou, China

**Keywords:** basal luteinizing hormone, BMI, central precocious puberty, girls, obesity

## Abstract

**Aim:**

To examined whether body mass index (BMI) affects basal gonadotropin secretion in premenarcheal girls with idiopathic central precocious puberty (ICPP).

**Methods:**

This retrospective single-center study involved girls diagnosed with ICPP. They were classified into underweight, normal weight, overweight, and obesity groups based on BMI-z scores. Clinical assessments included measurements of uterine and ovarian volumes and bone age (BA). Basal and gonadotropin-releasing hormone-stimulated luteinizing hormone (LH) and follicle-stimulating hormone (FSH) levels were measured. Correlation and partial correlation analyses were performed to explore relationships between BMI and aforesaid clinical assessments.

**Results:**

1077 girls (7.58 ± 0.91 years old) with ICPP were subdivided as normal weight (n=613), underweight (n=102), overweight(n=235), and obesity(n=115). Significant differences in clinical and hormonal characteristics were observed across weight groups. Underweight girls had lower basal LH as well as basal LH/FSH ratios compared to overweight and obesity groups. Overweight girls had higher basal LH than normal weight girls. Girls with obesity had no significant difference in basal LH compared to normal weight girls but had a positive within-group correlation between BMI and basal LH. Overall, BMI was positively correlated with basal LH, basal FSH, basal LH/FSH ratio, BA, BA-CA, uterine size and ovarian size, all of which persisted after adjusting for age and disease course.

**Conclusion:**

BMI is associated with basal LH in girls with ICPP, with distinct patterns across different weight groups. These findings highlight the importance of considering BMI when interpreting basal LH levels in the diagnosis of ICPP.

## 1 Introduction

Central precocious puberty (CPP) is the initiation of puberty before the age of 8 years in girls as a result of premature activation of the hypothalamo-pituitary-gonadal (HPG) axis. Girls with CPP have high luteinizing hormone (LH) levels and a high LH-to-FSH ratio for age, with gonadal function being driven by increased secretion of gonadotropin-releasing hormone (GnRH). Notwithstanding that the mechanism of puberty onset is unclear, the hallmark of early puberty is sleep-entrained increase in LH pulse frequency and amplitude with subsequent reduction of LH release during waking hours (1, 2).

Childhood obesity is a major global public health problem. Human and animal studies have corrobated that obesity modifies gonadotropin secretion. Serum LH concentration has been reported to be lower in both prepubertal and pubertal girls who are overweight or obese as compared with girls with normal weight (3). Several hypotheses have been proposed to explain the findings. In adults, increased body mass index (BMI) was associated with increased clearance of endogenous LH, and thus could result in lower serum LH concentration in individuals with greater BMI (4).

In girls studied via overnight sampling, obesity was associated with reduced LH pulse frequency during prepuberty and early puberty but increased frequency during later puberty (5). Prepubertal and early pubertal girls with obesity also had relatively low morning LH values and reduced LH pulse amplitude throughout puberty (6).

At present, several studies have analyzed the characteristics of LH peaks in CPP girls with obesity (7, 8). Insulin resistance increases both serum androgens and estrogens of ovarian and adrenal origin, which may reduce the peak LH response in children with obesity (9, 10).

Recent findings suggest that basal morning blood levels of gonadotropins can serve as indicators for CPP (11). Alterations in baseline blood LH, the rate of gonadal and sexual development, height, and the growth velocity change are key parameters in monitoring CPP progression (12). However, their utility as early diagnostic indicators in clinical practice is tentative. To date, there have been no studies concerning how BMI affects basal gonadotropins in CPP girls.

Hence, we retrospectively analyzed 1077 premenarcheal girls diagnosed with idiopathic central precocious puberty (ICPP), to clarify the impact of BMI on basal levels and ratios of LH and FSH.

## 2 Patients and methods

### 2.1 Study population

Retrospective review of a single-center study was carried out in 1077 girls who were admitted to our center for early breast development between January 2018 to April 2024. The exclusion criteria were as follows: 1) Girls diagnosed with CPP due to organic pathology; 2) CPP girls who had experienced menarche. In total, 1077 girls were included in the final analysis.

The study protocol was approved by the Medical Ethics Committee of Fuzhou Children’s Hospital of Fujian Medical University (Approval No. 201927), and was carried out in compliance with the Declaration of Helsinki.

### 2.2 Clinical assessment

Height and weight were measured by trained nurses. BMI-z scores (BMI-Z) were calculated based on reference values of Li Hui et al. (13). Secondary sexual examination was conducted by trained pediatric endocrinologists, including evaluation of breast development and distribution of pubic hair. Breast development was evaluated visually and by palpation. In overweight and obese girls, breast development was reconfirmed by ultrasound to distinguish true breast development from adipomastia. In the girls where Tanner stage for breast development was dissimilar, the breast that was more mature was documented for evaluation purposes. Bone age (BA) was based on left hand radiograph including phalanges of fingers, carpal bones and distal radius and ulna. BA was interpreted using the method of Tanner-Whitehouse 3 (TW3) (14). Ultrasound examinations were performed adhesives to conventional full bladder technique. Longitudinal (L), anteroposterior (AP), and transverse (T) diameters of the uterus as a whole, the corpus and cervix separately, and of the ovaries were measured. The formula for a prolate ellipsoid (V = L × AP × T × 0.5233) was used to calculate the volume (V) of both the uterus and ovaries (15).

Baseline blood samples were taken before intravenous (IV) administration of gonadorelin (2.5μg/kg, maximum 100 μg). The samples of LH and FSH were drawn at 30, 60, 90 and 120 min after the IV GnRH. LH and FSH were measured by chemiluminescence (Siemens Healthcare Diagnostics, Los Angeles, CA, USA). The lower limit of sensitivity for LH and FSH is 0.1 IU/L. The intra-assay coefficient varies from 2.6% to 8.5% whereas the inter-assay coefficient varies from 3.7% to 11.9%.

### 2.3 Diagnosis and weight classification

The diagnosis of CPP depends upon the combination of clinical manifestations and peak LH response to the GnRH stimulation test. The cutoff level for the diagnosis of CPP was peak LH ≥ 5IU/L (10).

Subjects were classified according to their BMI-z scores (BMI-Z): normal weight (BMI-Z for age between -1SD to 1SD), overweight (BMI-Z for age between 1SD to 2SD), obesity (BMI-Z for age ≥2SD), and underweight (BMI-Z for age <-1SD).

### 2.4 Statistical analysis

Statistical analysis was performed using SPSS version 26.0 (IBM SPSS, Chicago, IL). For the normal distribution parameters (CA, height, weight, BMI, BA, Uterine Size, Ovary Size) tested by the Kolmogorov–Smirnov test, one-way analysis of variance (ANOVA) was performed with the *post hoc* test of Tukey for multiple comparisons. For the skewed parameters (basal LH, basal FSH, ratio of basal LH/FSH, peak LH, peak FSH, and ratio of peak LH and FSH), the Kruskal–Wallis tests were used and thereafter Mann–Whitney tests were used for pairwise comparisons. Correlation analysis was conducted using Pearson’s correlation and partial correlation. Statistical significance was defined as *P* < 0.05. Results were presented as mean ± SD, or median (interquartile range) when appropriate.

## 3 Results

### 3.1 Basic clinical features and hormone profiles stratified by weight status

This study included 1077 girls diagnosed with ICPP, whose age ranged from 4.5 to 10.58 years, with a mean of 7.58 ± 0.91 years. Based on BMI-Z, the study population (n=1077) were subdivided as normal weight (n=618), underweight (n=104), overweight(n=239), and obesity(n=116). Clinical characteristics are shown in **Table 1**.

**Table 1:** Clinical characteristics of study subjects according to BMI (mean ± SD).

Parameter	Underweight	Normal weight	Overweight	Obesity	*P*
n	104	618	239	116	
CA (years)	7.26 ± 1.01	7.58 ± 0.88	7.69 ± 0.90	7.67 ± 0.90	0.001
Onset age (years)	6.74 ± 0.92	6.93 ± 0.75	6.94 ± 0.79	6.85 ± 0.80	0.097
Course (years)	0.52 ± 0.65	0.64 ± 0.65	0.75 ± 0.64	0.82 ± 0.70	0.001
Height (cm)	123.51 ± 8.88	127.36 ± 7.06	130.55 ± 7.47	131.83 ± 7.60	<0.001
Weight (kg)	20.17 ± 3.12	24.93 ± 3.46	30.68 ± 4.17	35.96 ± 5.68	<0.001
BMI (kg/m2)	13.14 ± 0.55	15.29 ± 0.92	17.91 ± 0.79	20.56 ± 1.50	<0.001
BMI SDS	-1.53 ± 0.51	0.05 ± 0.56	1.44 ± 0.29	2.47 ± 0.42	<0.001
BA (years)	8.68 ± 1.79	9.51 ± 1.49	10.04 ± 1.44	10.31 ± 1.32	<0.001
BA-CA (years)	1.44 ± 1.22	1.93 ± 1.07	2.36 ± 0.99	2.65 ± 0.89	<0.001
Uterine size (cm^3^)	1.37 ± 0.64	1.78 ± 1.25	2.17 ± 1.88	2.02 ± 1.70	<0.001
Left ovary size (cm^3^)	1.72 ± 0.78	1.97 ± 0.85	2.15 ± 0.94	2.14 ± 1.00	<0.001
Right ovary size (cm^3^)	1.83 ± 1.08	2.00 ± 0.86	2.23 ± 1.07	2.14 ± 1.03	0.001

There was no statistically significant difference in the age of onset among the groups (all *P*>0.05). The chronological age(CA) and left ovary size of the underweight group was significant smaller than the other three cohorts(all *P* < 0.05). The course and uterine size of underweight group was significant smaller than the overweight and obesity groups (all *P* < 0.05). There was no statistical difference in BA and BA-CA between the overweight and the obesity groups (both *P*>0.05). The uterine size of the overweight group was significant larger than the other three groups(all *P* < 0.05).

**Figure 1:**
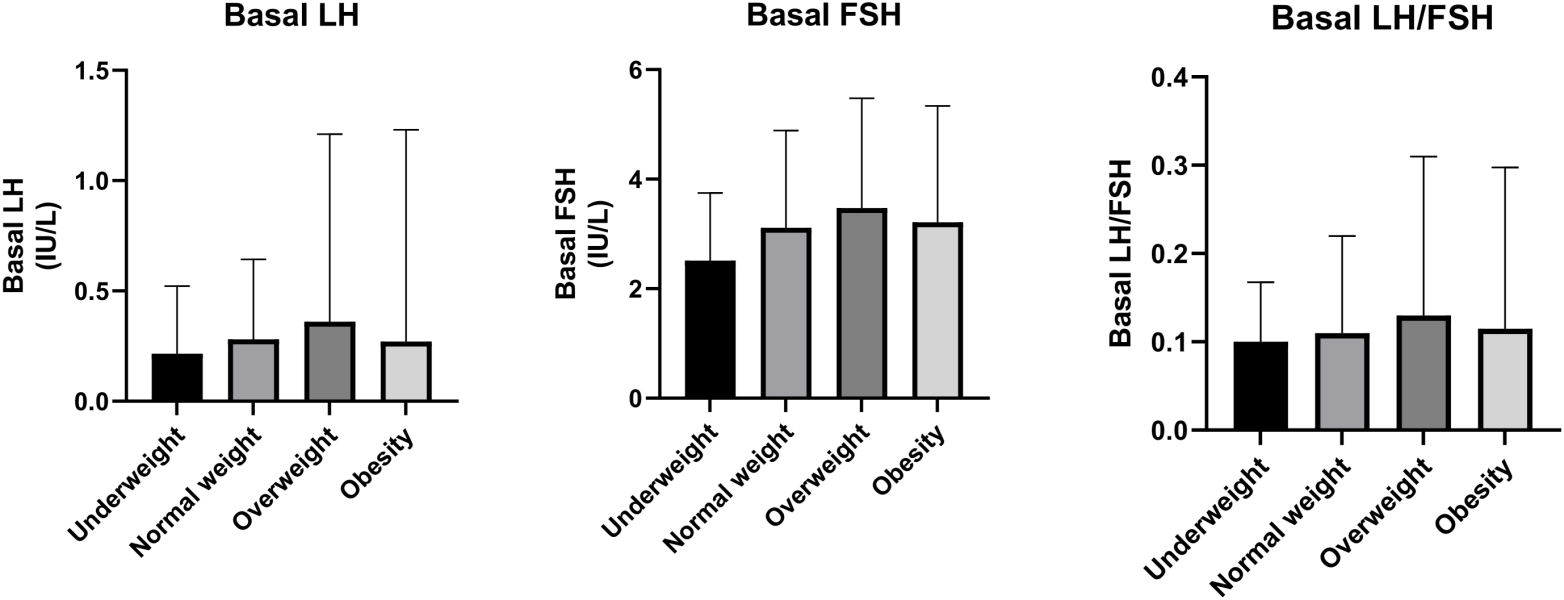
Bar charts of sex hormone levels in ICPP girls with different weight statuses [median (25th percentile, 75th percentile)].

As for hormonal values, the basal LH and ratio of basal LH/FSH of the underweight group was significantly lower than the overweight and obesity groups (both *P* < 0.05). The basal FSH of the underweight group was significantly lower than the overweight group (*P* < 0.05). The basal LH of overweight group was significantly higher than the normal weight group (*P* < 0.05).There was no statistical difference in basal LH between the normal weight group and the obesity group (*P*>0.05). Hormonal values are shown in **Table 2**, **Figure 1**.

**Table 2:** Hormonal values stratified by weight status [median (25th percentile, 75th percentile)].

Parameter	Underweight	Normal weight	Overweight	Obesity	*P*
n	104	618	239	116	
Basal LH (IU/L)	0.22(0.14,0.52)	0.28(0.15,0.64)	0.36(0.17,1.21)	0.27(0.14,1.23)	<0.001
Basal FSH (IU/L)	2.52(1.85,3.75)	2.66(1.84,3.96)	3.06(2.06,4.51)	2.70(1.65,4.23)	0.020
Basal LH/FSH	0.10(0.06,0.17)	0.11(0.07,0.22)	0.13(0.08,0.31)	0.12(0.08,0.30)	0.001
Peak LH (IU/L)	8.76(6.69,12.90)	9.74(6.95,17.83)	10.90(7.33,19.80)	9.56(7.04,18.38)	0.010
Peak FSH (IU/L)	14.90(10.95,20.50)	13.10(10.20,17.10)	13.20(10.50,17.30)	12.95(9.82,17.50)	0.030
Peak LH/FSH	0.61(0.43,1.00)	0.81(0.54,1.45)	0.89(0.54,1.57)	0.88(0.54,1.41)	0.005

### 3.2 The correlation between BMI and the gonadotropins, BA and pelvic ultrasound

Correlation analysis for the 1077 cases found that BMI had a positive relationship both with basal LH, basal FSH, the basal ratio LH/FSH, BA, BA-CA, uterine size and ovary size (all P  < 0.05).

Correlation analysis for the underweight subjects found that there was no correlation between BMI and development-related indicators (all *P*  > 0.05).

Normal weight subjects had a positive relationship of BMI with basal LH, basal FSH, the ratio basal LH/FSH, BA, BA-CA, uterine size and ovary size (all *P*  < 0.05).

Correlation analysis for the overweight subjects disclosed that BMI had a positive relationship with basal LH, the ratio basal LH/FSH, BA, uterine size and ovary size (all *P*  < 0.05).

For girls with obesity, BMI had a positive relationship with basal LH, the ratio basal LH/FSH, BA, uterine size and ovary size (all *P*  < 0.05). Data are shown in **Table 3**.

**Table 3:** Pearson’s correlation table on BMI and sexual development-related indicators stratified by weight status.

Index	Parameter	Total	Underweight	Normal weight	Overweight	Obesity
n		1077	104	618	239	116
Basal LH	R	0.199**	-0.005	0.186**	0.130*	0.209*
	*P*	<0.001	0.963	<0.001	0.045	0.025
Basal FSH	R	0.091**	0.031	0.115**	0.028	-0.001
	*P*	0.003	0.754	0.004	0.670	0.992
Basal LH/FSH	R	0.182**	0.012	0.211**	0.140*	0.268**
	*P*	<0.001	0.907	<0.001	0.031	0.004
BA	R	0.345**	0.071	0.238**	0.423**	0.350**
	*P*	<0.001	0.481	<0.001	<0.001	<0.001
BA-CA	R	0.317**	0.048	0.165**	0.136*	0.071
	*P*	<0.001	0.636	<0.001	0.036	0.446
Uterine size	R	0.220**	0.175	0.238**	0.239**	0.325**
	*P*	<0.001	0.085	<0.001	<0.001	<0.001
Left ovary size	R	0.203**	0.195	0.196**	0.212**	0.320**
	*P*	<0.001	0.055	<0.001	0.001	0.001
Right ovary size	R	0.185**	0.197	0.186**	0.216**	0.329**
	*P*	<0.001	0.051	<0.001	0.001	<0.001

*P < 0.05; **P < 0.01.

### 3.3 Partial correlation analysis

Considering the differences in age and course among the groups, a partial correlation analysis after adjusting for age and course was conducted. BMI had a positive relationship with basal LH, the ratio basal LH/FSH, BA, BA-CA, uterine size and ovary size (all *P*  < 0.05). Data are shown in **Table 4**.

**Table 4:** Partial correlation analysis of BMI and sexual development-related indicators after adjusting for age and course in general population.

Parameter	R	P
Basal LH	0.127*	<0.001
Basal FSH	0.042	0.192
Basal LH/FSH	0.112*	<0.001
BA	0.275*	<0.001
BA-CA	0.275*	<0.001
Uterine size	0.132*	<0.001
Left ovary size	0.113*	<0.001
Right ovary size	0.095*	0.003

*P < 0.01.

## 4 Discussion

Basal LH constitutes a pivotal benchmark for evaluating the activation of the HPG axis in girls with CPP (16–18). Extensive investigations have been reported over the years to explore the reliable role of basal gonadotropin levels in the diagnosis of CPP (19–21). Recent clinical guidelines advocate for a diminished reliance on GnRH/Gonadotropin-releasing hormone analog (GnRHa) stimulation tests, instead prioritizing basal LH as a trustworthy biochemical marker for CPP diagnosis (22). Nevertheless, limitations exist owing to the pulsatile secretion pattern of LH and its susceptibility to BMI as well as assay methodologies (23).

The present study involving 1077 CPP girls identified distinct patterns of basal LH levels across different weight strata, thereby shedding light on the intricate interplay between BMI and gonadotropin secretion. Our findings revealed a positive correlation between basal LH levels and BMI. Statistical analyses indicated that among premenarcheal girls with ICPP, basal LH levels as well as the basal LH/FSH ratio were significantly lower in individuals with obesity compared to their overweight counterparts, while being marginally higher than in CPP girls of normal weight. These data are consistent with the possibility that excessive adiposity may paradoxically exert a subtle suppressive effect on HPG axis function during early puberty (7).

Overall, the study underscores that BMI exhibits a positive correlation with basal LH levels in girls with ICPP, with distinct patterns observable across disparate weight groups. The role of adipose-derived leptin, which is elevated in obesity, may impact the onset of puberty. Ir has been reported that Low-dose leptin initiates the central network that regulates gonadotropin secretion, whereas high concentrations of leptin in obese individuals may exert an inhibitory effect on the gonads (24).

Girls categorized as underweight displayed significantly lower basal LH levels and basal LH/FSH ratios in comparison to overweight and obesity groups. Their basal FSH levels were also lower than those in the overweight group, suggesting a relatively diminished activation of the HPG axis in this subgroup. A plausible explanation for the lower LH levels in the underweight group could be their shorter disease duration. Due to reduced subcutaneous fat, breast development in these girls may be more conspicuous to parents at an earlier stage, prompting earlier medical consultation. Thus, resulting in a shorter duration of puberty at the time of diagnosis. Correlation analysis further revealed that among underweight subjects, there was no correlation between BMI and development-related indicators, which is consistent with their lower basal gonadotropin profiles.

In normal weight girls, basal LH levels were intermediate. Notably, their basal LH was significantly lower than that in the overweight group, but similar to the obesity group. Correlation analysis found a positive relationship between BMI and basal LH, as well as with basal FSH, the basal LH/FSH ratio, and sexual development indicators such as uterine and ovarian volumes in normal weight group, reflecting a coordinated association between adiposity and HPG axis activity in normally weighted ICPP girls.

Overweight girls exhibited significantly higher basal LH levels compared to the normal weight group which may indicate an amplified HPG axis response associated with increased adiposity. Specifically, we hypothesize that adipose tissue can secrete leptin, and fat accumulation in overweight girls leads to elevated leptin levels. Leptin can directly activate gonadotropin-releasing hormone (GnRH) neurons in the hypothalamus, increasing the frequency and amplitude of GnRH secretion and thereby elevating LH levels (23). Further investigation may unravel the underlying mechanism, such as leptin,cytokines, adiponectin, steroid hormones.

Interestingly, despite higher BMI, girls with obesity did not exhibit a statistically significant difference in basal LH levels compared to the normal weight group. However, correlation analysis confirmed a positive relationship between BMI and basal LH in subjects with obesity. This seemingly paradoxical observation—whereby girls with obesity do not have higher basal LH levels than their normal weight peers but display a positive BMI-related trend within their group—may be attributed to undefined complex regulatory mechanisms. For instance, increased estrogen and androgen levels in obesity may exert partial inhibitory effects on LH secretion (9, 10), which could counterbalance the stimulatory effects of adiposity on the HPG axis.

The observed patterns of basal and stimulated LH in ICPP girls across weight groups are likely mediated by interconnected mechanisms, including, but not limited to, insulin resistance-driven hyperandrogenism/hyperestrogenism, estrogenic negative feedback on the pituitary, leptin resistance, and potentially altered LH clearance (24). These pathways collectively account for the link between obesity and blunted GnRH-stimulated LH peaks, despite the positive association between BMI and basal LH. Furthermore, the persistence of a positive correlation between BMI and basal LH levels, even after adjusting for age and disease course, validates our hypothesis regarding the influence of disease duration (that is, earlier detection of breast tissue) in the underweight group.

The comprehensive mechanism underlying the significant influence of BMI on LH and FSH secretion in girls with precocious puberty remains incompletely understood. The current research provides limited yet meaningful insights into the hormonal factors involved in this dynamic developmental process. Consequently, further research is required to explore adipokinetic hormones and their receptors. Our results indicated that basal LH values in girls with obesity were lower than those in overweight girls. Therefore, in the assessment of CPP girls with obesity or overweight, it is necessary to consider the impact of BMI on pubertal hormone concentrations.

McCartney et al’s study offered valuable perspectives on the impact of obesity on LH secretion patterns during pubertal maturation in girls. The findings demonstrated that prepubertal and early pubertal girls with obesity exhibited reduced LH pulse frequency and amplitude, with an absence of the nocturnal increases typically observed in their non-obese counterparts. In contrast, girls with obesity in later pubertal stages displayed elevated LH pulse frequency but reduced amplitude, a pattern potentially driven by hyperandrogenemia. These alterations underscored the complex interplay between obesity, LH secretion dynamics, and androgen excess, combinations of which could disrupt normal pubertal progression (5). Another study reported that the sleep-related increase in gonadotropins was attenuated in healthy, overweight girls in the early stage of puberty (25).

The limitation of this study is that variations in bona fide breast adipose tissue across weight groups may bias Tanner developmental stage assessment. This may render developmental stages across groups incomparable, confounding basal LH and pubertal progression interpretations. The cross-sectional study design limits the ability to clarify temporal relationships between BMI, developmental stage, and basal LH. Subsequent longitudinal studies will compare basal LH in different BMI groups at the same developmental stage to reduce timing-related bias and better assess their independent association.

Additionally, given the limitations of BMI, which cannot accurately reflect the levels of fat mass and lean mass in the body, we expect that prospective studies can be carried out in the future. Body composition analyzers and other tools can be used to accurately measure fat mass and lean mass, so as to further explore the specific correlation between them and LH levels as well as the underlying regulatory mechanisms, thus revealing the role of body composition in the occurrence and development of CPP more deeply.

Hormones exhibit significant seasonal variations, with similar fluctuations observed in mood and emotional responses (26). The most notable seasonal changes occur in growth hormone levels. However, there are also considerable seasonal differences in brain neurochemistry that could affect eating habits and BMI. Furthermore, tt has been reported that WFS1 gene influences hormones, emotions, and mental health (27). WFS1 is directly involved in hormone secretion and mood regulation. As this was a retrospective study, relevant data were lacking for further analysis of the impact of season or WFS1 gene on basal LH and FSH levels, and this aspect will be refined in subsequent research.

These findings emphasize the importance of considering BMI when interpreting basal LH levels in the diagnosis of CPP, as previously suggested by guidelines highlighting the sensitivity of LH to BMI (22).

## Data Availability

The original contributions presented in the study are included in the article/Supplementary Material. Further inquiries can be directed to the corresponding author.
